# Detecting Subtle Changes in Visuospatial Executive Function and Learning in the Amnestic Variant of Mild Cognitive Impairment

**DOI:** 10.1371/journal.pone.0021688

**Published:** 2011-07-14

**Authors:** Kathryn V. Papp, Peter J. Snyder, Paul Maruff, Jennifer Bartkowiak, Robert H. Pietrzak

**Affiliations:** 1 Department of Psychology, University of Connecticut, Storrs, Connecticut, United States of America; 2 Centre for Neuroscience, University of Melbourne, Parkville, Victoria, Australia; 3 CogState, Ltd., Melbourne, Victoria, Australia; 4 Department of Clinical Neurosciences, Warren Alpert Medical School of Brown University & Lifespan Hospitals System, Providence, Rhode Island, United States of America; 5 National Center for Posttraumatic Stress Disorder, VA Connecticut Healthcare System, West Haven, Connecticut, United States of America; 6 Department of Psychiatry, Yale University School of Medicine, New Haven, Connecticut, United States of America; University of British Columbia, Canada

## Abstract

**Background and Purpose:**

Amnestic mild cognitive impairment (aMCI) is a putative prodromal stage of Alzheimer's disease (AD) characterized by deficits in episodic verbal memory. Our goal in the present study was to determine whether executive dysfunction may also be detectable in individuals diagnosed with aMCI.

**Methods:**

This study used a hidden maze learning test to characterize component processes of visuospatial executive function and learning in a sample of 62 individuals with aMCI compared with 94 healthy controls.

**Results:**

Relative to controls, individuals with aMCI made more exploratory/learning errors (Cohen's d = .41). Comparison of learning curves revealed that the slope between the first two of five learning trials was four times as steep for controls than for individuals with aMCI (Cohen's d = .64). Individuals with aMCI also made a significantly greater number of rule-break/error monitoring errors across learning trials (Cohen's d = .21).

**Conclusions:**

These results suggest that performance on a task of complex visuospatial executive function is compromised in individuals with aMCI, and likely explained by reductions in initial strategy formulation during early visual learning and “on-line” maintenance of task rules.

## Introduction

In older adults, amnestic mild cognitive impairment (aMCI) is classified on the basis of objective evidence of specific and relatively large (i.e. >1 to 1.5 standard deviations) impairment in episodic memory, self- or informant-reported problems in memory but intact activities of daily living and mood [1], [2]. Neurobiological evidence is mounting that aMCI represents the earliest stages of Alzheimer's disease (AD) in the majority of patients [3], [4], [5]. Neuropsychological models of aMCI emphasize specific impairment in episodic verbal memory such that the presence of impairment in additional cognitive domains requires an alternative diagnosis such as multiple-domain MCI, whose pathophysiological and prognostic models are less clear compared with aMCI [6], [7], [8].

Given that mild AD is typically characterized by impairment in memory, the importance of specific memory impairment to both clinical models of aMCI is clear. However, there is now growing evidence that executive functions may also be reduced in individuals who meet clinical criteria for aMCI, albeit at a magnitude not large enough to satisfy criteria for multiple-domain MCI. For example, Brandt and colleagues found that individuals with aMCI were worse on measures of planning/problem solving and working memory, but not judgment (e.g. Iowa Gambling Test), relative to healthy controls [9]. The magnitude of these impairments relative to controls was small to moderate (e.g., Cohen's *d* = 0.46 for planning/problem solving and Cohen's *d* = .49 for working memory). As would be expected, individuals with multiple-domain aMCI had more pronounced reductions in planning/problem solving and working memory than individuals with single domain aMCI. Taken together, results of these studies, coupled with findings suggesting that executive dysfunction is related to functional impairment in older adults [10], [11], underscore the importance of understanding the nature of executive dysfunction in aMCI. In fact, some researchers have concluded that only when executive function becomes impaired should an MCI patient be considered to have prodromal AD [12], [13].

While the magnitude of impairments in aspects of executive function in patients with MCI is typically small, it is important for three reasons. First, elucidation of aspects of executive function that are reduced in aMCI may show how other cognitive systems in addition to memory may break down in the dementia prodrome. For example, individuals with aMCI who have executive dysfunction may have difficulty organizing material at the level of encoding, strategically retrieving information, and overcoming the effects of interference [14], [15], [16]. Second, impairment in aspects of executive function may provide insight into bases for memory impairment. For example, a recent study by Chang and colleagues found that individuals with MCI who scored higher on measures of executive function (Trail Making Test and Digits Backward) performed better on a measure of episodic verbal memory compared to individuals with MCI who scored lower on measures of executive function [17]. One explanation for this association is that executive function may help to facilitate cognitive processes involved in verbal learning (e.g. use of strategies such as semantic clustering) and may help explain impairment on measures of verbal and visuospatial learning and memory in individuals with MCI. Finally, given that performance on tasks of complex executive function requires the coordination of multiple cognitive operations including memory, poor performance on these tasks may merely be the consequence of the memory dysfunction that warranted the clinical classification (i.e., forgetting test rules). That said, the relatively moderate nature of the executive reductions found in individuals with aMCI may also reflect very early changes in prefrontal cortical systems, which are hypothesized to be necessary for normal executive functions.

Many of the tasks used to assess complex executive function in aMCI are limited in the extent to which performance can be understood in terms of component cognitive operations such as aspects of executive function and learning/memory [18]. Nevertheless, recent studies using hidden pathway maze learning to study executive function [19], [20] have shown that the computerized administration and scoring of these tasks (e.g., the Groton Maze Learning test [GMLT]) allows analysis of the extent to which learning/memory and executive processes contribute to overall performance [19], [20], [21]. Hidden pathway maze learning as assessed by the GMLT requires individuals to learn the location of a complex maze pathway hidden beneath a 10x10 grid of tiles over successive learning trials. Individuals are trained on a set of rules that facilitate search prior to attempting the maze and during performance these rules are reinforced by error signals made whenever a choice made contravenes one of these rules. On the initial learning trial, individuals locate the pathway using a trial-and-error search strategy conducted within the context of the pre-learned rules. As the pathway is found across repeated trials, a representation of the pathway location in memory strengthens and can then be integrated with the application of the maze rules to facilitate navigation of the pathway. Thus, optimal performance on this task reflects the ability of individuals to combine rule application with representations of a hidden maze pathway in spatial memory. Further, the errors made across successive learning trials can be classified as reflecting contravention of the maze rules (i.e., rule break errors) or as errors in remembering the maze pathway (i.e., exploratory errors).

There is growing evidence for the independence of these two error types. For example, challenge with scopolamine, a cholinergic antagonist, in healthy adults is associated with large-magnitude performance deficits on the Groton Maze Learning Test in healthy older adults. Importantly, the deleterious effects of scopolamine were more pronounced for rule break/error monitoring errors than for exploratory/learning errors, suggesting that inhibition of cholinergic neurotransmission may affect executive functions to a greater extent than spatial memory functions [21]. In children with attention-deficit/hyperactivity disorder (ADHD), poor performance on the GMLT arises because these children make more rule-break errors than age-matched children, whereas no differences between children with ADHD and controls are observed for exploratory errors. Further, in children with ADHD, stimulant medication reduced rule break errors but did not alter rates of exploratory errors [22]. In contrast, in healthy adults, poor performance on the GMLT under low-level alcohol intoxication was due predominately to an increase exploratory errors but not rule break errors, suggesting that modulation of gamma aminobutyric acid (GABA) neurotransmission affected spatial learning more so than executive function [23]. Taken together, these data suggest that hidden pathway maze learning may provide a useful neuropsychological probe to understand the nature of subtle reductions in aspects of visuospatial executive function and learning that occur in aMCI.

The aim of this study was to examine complex executive function and learning in a well-characterized sample of individuals with aMCI using a hidden pathway maze learning task and to determine the extent to which impairment on this measure of complex executive function reflects the contribution of executive or spatial learning processes. Given that adults with aMCI have substantial impairments in episodic memory, we hypothesized that poor performance on the Groton Maze Learning Test in older adults with aMCI would reflect predominantly dysfunction of component spatial memory processes.

## Materials and Methods

### Participants

Participants were pooled from the control arms of four separate pharmaceutical trials occurring between 2003–2007 with sites in Syracuse (New York, USA), Toronto (Ontario, Canada), Austin (Texas, USA) and Melbourne (Victoria, Australia). They were recruited through newspaper advertisements for “studies on human memory” at the respective sites. The protocol was approved by the Western Institutional Review Board for the US sites and by the Ethics Review Board of the University of Brisbane for the Australian site. All participants provided written informed consent prior to participating in this study. For all four of these studies, participant inclusion and exclusion criteria as well as study recruitment and procedures were identical (described below). The goal of recruitment for these trials was to obtain an exceptionally well-characterized and ‘clean’ sample of patients with aMCI.

Data were collected for a total of 156 elderly adults between the ages of 55 and 90 (Mean age = 68.5; 43.6% male), 62 of whom were diagnosed with amnestic mild cognitive impairment (aMCI) and 94 of whom were classified as healthy elderly (HE). Demographic characteristics of these groups are shown in Table 1.

**Table 1:** Demographic characteristics of healthy elderly and aMCI groups.

	Healthy Elderly	aMCI	Statistical test
N	92	59	
Age	67.4 (SD = 8.8)	69.9 (SD = 8.1)	t(149) = 1.81, p = .072
Sex (%male)*	n = 32 (34.8%)	n = 32 (54.2%)	χ^2^(1) = 5.57, p = .018
Years of education	12.9 (SD = 3.9)	12.1 (SD = 3.0)	t(149) = 1.67, p = .10

*Note*: aMCI = Mild cognitive impairment-amnestic subtype; SD = standard deviation.

*Groups differ, p<.05.

The following inclusion criteria for participants were followed in all four studies: 1) all participants were physically healthy as defined by no clinically relevant abnormalities identified by a detailed medical history and full physical examination (including blood pressure and heart rate measurement, 12-lead EEG and clinical laboratory tests); 2) all participants obtained a head CT or MRI within 12 months of testing, which showed no evidence of infection, infarction, or other focal lesions that could result in dementia; 3) all participants scored 12 or less on the 17-item Hamilton Depression Rating Scale; 4) all participants scored between 24 and 30 on the Mini-Mental State Exam; 5) all participants obtained scores of no more than .5 on the Clinical Dementia Rating Scale [24] and a score of at least .5 on the Memory Score Box [25]; and 6) all participants presented with general cognitive and functional performance sufficiently preserved such that the site physician could not make a diagnosis of Alzheimer's disease at the time of the screening visit. Criteria for those in the aMCI group, in addition to those outlined above, include 1) participants diagnosed with aMCI reported memory complaints and memory difficulties verified by an informant and 2) participants diagnosed with aMCI exhibited abnormal memory function documented by scoring at least 1 standard deviation below the education- and age-adjusted cut-off on the Logical Memory II subscale (Delayed Paragraph Recall) from the Wechsler Memory Scale-Revised with preserved performance in other cognitive domains (within 1 standard deviation of age and education adjusted means). Scores on cognitive tests used to classify groups are shown in Table 2.

**Table 2:** Neuropsychological tests used to classify Healthy Elderly and aMCI groups.

	Mean (SD)	
	Healthy Elderly	aMCI
Mini Mental State Exam*+	29.2 (1.01)	27.7 (1.35)
Rey Osterrieth Complex Figure Copy*	33.0 (2.43)	31.0 (4.75)
ROCF 3 min delay*	19.0 (6.14)	17.0 (5.39)
ROCF 30 min delay*+	18.0 (4.59)	13.0 (4.00)
Wechsler Memory Scale Logical Memory Delayed*+	12.0 (2.11)	8.0 (2.57)
Rey Auditory Verbal Learning List A- Immediate*+	10.0 (2.75)	6.0 (2.93)
RAVLT List A- Delayed*+	10.0 (2.76)	6.0 (3.24)

*Groups differ, p<.05, +aMCI group impaired (mean performance more than 1 SD below healthy elderly mean performance), SD = standard deviation, aMCI = Amnestic mild cognitive impairment.

Participants were excluded if they displayed 1) evidence or history of clinically significant hematological, renal, endocrine, pulmonary, gastrointestinal, cardiovascular, hepatic, psychiatric, neurological, or allergic disease; 2) uncontrolled hypertension; 3) current depression or a history of major depression or another major psychiatric disorder as described in the Diagnostics and Statistics Manual- Version IV (DSM-IV) within the past 2 years; 4) history of regular alcohol consumption exceeding 7 drinks/week for women or 14 drinks/week for men within 6 months of screening or 5) history of substance abuse or dependence within the past 2 years (DSM-IV criteria). Study recruits were excluded if they were using the following medications: anti-Parkinsonian medications or anti-convulsants within 2 months prior to screening, neuroleptics or narcotic analgesics, long-acting benzodiazepines or barbiturates, warfarin (Coumadin), antidepressants, nitrates, phosphodiesterase inhibitors, sympathomimetic agents for asthma control, ocular glaucoma drops, centrally active beta-blockers, narcotics, methyldopa and clonidine, or medications with significant cholinergic or anticholinergic side effects within 4 weeks prior to screening, short-acting anxiolytics or sedative hypnotics more frequently than 2 times per week within 4 weeks prior to screening, or systemic corticosteroids within 3 months prior to screening.

### The Groton Maze Learning Test (GMLT)

**Figure 1:**
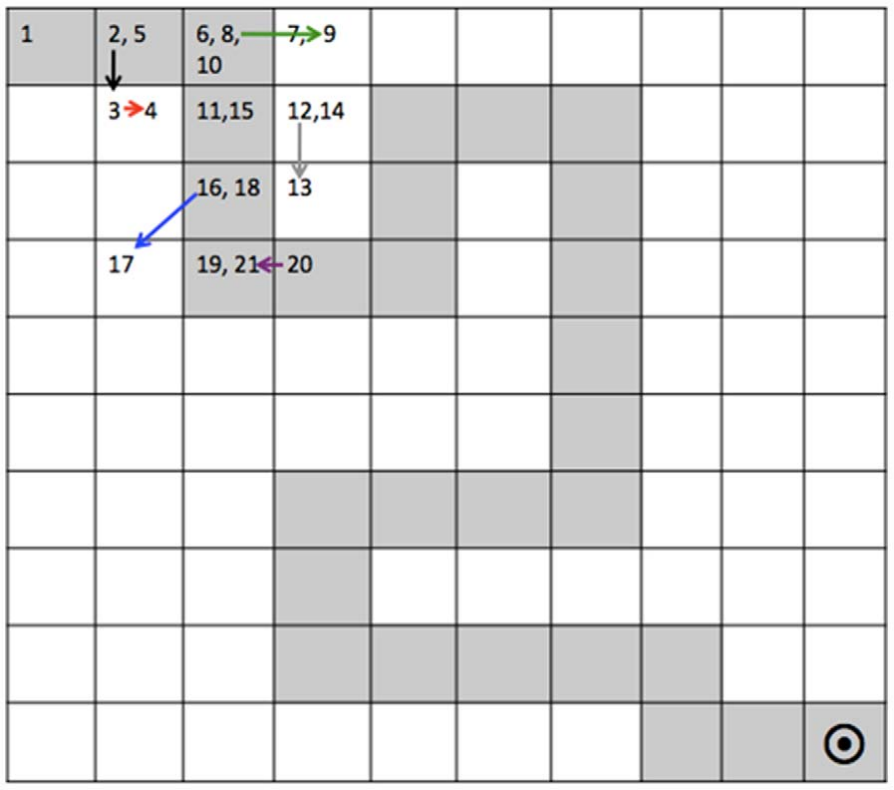
The Groton Maze Learning Test: Stimulus and Examples of Error Types. Tiles shaded in gray represent one of the hidden maze pathways on the GMLT; numbers indicate order of moves for a sample participant; Move 2 to 3 (shown in black): exploratory error; Move 3 to 4 (shown in red): rule-break error (tapping on same tile twice); Move 8 to 9 (shown in green): perseverative error; Move 12 to 13 (shown in grey): rule break error (not moving back to the last correct tile); Move 16 to 17 (shown in blue): rule break error (no moving diagonally); Move 20 to 21 (shown in purple): rule break error (moving backwards along the maze).

The GMLT, originally developed by one of the authors (P.J.S.), has been well-described elsewhere [26]. Briefly, this test begins with a “timed chase test” and is then followed by the hidden maze learning task. The timed chase test is a measure of simple visuomotor processing speed that uses the same touchscreen input device and spatial array of grey “tiles” as the maze test, but without the maze-learning component. Participants are shown a 10×10 grid of tiles on a computer touch screen (see Figure 1) and they are asked to ‘chase the target,’ a blue tile that moves erratically around the grid. As the target moves, the participant ‘chases’ it by tapping on the tiles one at a time (essentially, playing a game of ‘follow the leader’). They are instructed to follow the following rules: 1) no diagonal moves; 2) no skipping tiles; 3) after an incorrect move, return to the last correct tile, and 4) do not tap twice on the same tile. Once completing the untimed practice task, the number of correct moves made during a second 30-sec trial is used as an index of visuomotor speed. This metric is used as a covariate to control for the potentially confounding influence of visuomotor processing speed on GMLT performance.

To complete the hidden maze task, the participant must learn a hidden pathway of 28 moves and 11 turns through the 10×10 grid from the top left corner to a finish marker in the bottom right corner. They must follow the same four rules listed above. Message bars and a musical tone indicate correct versus incorrect moves, and the participant is instructed to either ‘go on’ or move back to the last correct tile. Participants complete the trial five times, in rapid uccession, to learn the same hidden path. Several error types are recorded, including ‘legal’ or exploratory errors (errors made while following rules; measure of maze memory), perseverative errors (an exploratory error made repeatedly on a consecutive trial), and “rule-break errors” (defined as errors made in contravention of standard GMLT rules: i.e., no moving diagonally, skipping tiles, failing to return to the last correct move after an error, do not tap twice on the same tile). These measures are generated for the five learning trials, as well as for the delayed recall trial. A savings scores, which is the difference in total moves between the fifth learning trial and the delayed recall trial is also computed [20]. All mazes contain an equal number of moves with no tiles revisited in a single maze. Each participant completed a randomly generated maze. To control for ‘task familiarity effects,’ all participants completed two GMLT practice sessions using different mazes within one week prior to their baseline exams. The GMLT is available for both research and clinical use, by contacting the test vendor, CogState, Ltd., at www.cogstate.com.

### Data Analysis

Demographic characteristics of the aMCI and HE groups were compared using independent-samples t-tests or chi-square tests. GMLT outcome measures were computed according to previously defined operationalizations of component cognitive processes assessed by the task [27]. Non-normally distributed measures (e.g., perseverative and rule-break errors) were transformed using logarithmic (log10) transformations prior to analysis. GMLT performance was analyzed using analyses of covariance (ANCOVA) with group (HE vs. aMCI) and demographic characteristics that differed between groups entered as covariates. A univariate ANCOVA was conducted to analyze performance on the timed chase test of the GMLT, with group (HE vs. aMCI) entered as a fixed factor and age and sex as covariates. Multivariate ANCOVAs were conducted to analyze performance on GMLT measures of exploratory, perseverative, and rule-break errors, differences in exploratory errors, and learning slopes; age, sex, and timed chase test scores were entered as covariates in this analysis; a separate ANCOVA was conducted with delayed recall measures entered as dependent variables. Cohen's d [28] values were computed to estimate magnitudes of group differences.

## Results

As shown in Table 1, age did not differ between groups. The groups differed with respect to sex, with slightly more males in the aMCI group. Age and sex were entered as covariates in analyses of GMLT scores.

A univariate ANCOVA revealed that the aMCI group made fewer moves per second on the timed chase test (28.99±.79 vs. 33.89±.79; F(1,146) = 15.53, p<.001, d = .69) compared with healthy controls. Greater age was also associated with performance on the timed chase test (F(1,146) = 136.63, p<.001; r = −..69, p<.001), but sex (F(1,146) = .95, p = .33), and the interaction of group x sex (F(1,146) = .00, p = .95, were not significant. Accordingly, to control for the effect of differences in visuomotor processing speed, timed chase test scores were entered as a covariate in a multivariate analysis of GMLT performance measures.

A MANCOVA of GMLT learning measures revealed a significant overall effect of group [F(5,143) = 2.86, p = .012]; age [F(5,143) = 3.67, p = .004] was also significantly associated with GMLT learning measures, but sex [F(6,143) = 1.23, p = .30], timed chase test scores [F(5,143) = .57, p = .72], and interactions of group x age [F(5,143) = 1.86, p = .11], group x sex [F(5,143) = .22, p = .97], and group x timed chase test scores [F(5,143) = 2.11, p = ..07] were not significant. As shown in Table 3, the aMCI group made more exploratory and rule-break errors than the healthy control group, and differed significantly on a measure of the learning slope from trial 1 to 2. The groups did not differ with respect to perseverative errors.

**Table 3:** Means and standard errors for Groton Maze Learning Test (GMLT) outcome measures in Healthy Elderly and aMCI groups.

	Healthy Elderly	aMCI	Statistical tests	Cohen's *d*
*Learning Trials*				
Exploratory Errors*	56.13 (1.98)	63.87 (2.39)	F(1,145) = 5.84, p = .017	.41
Perseverative Errors	4.84 (.54)	5.70 (.65)	F(1,145) = 2.79, p = .097	.17
Rule-break errors*	2.31 (.38)	3.06 (.45)	F(1,145) = 4.40, p = .038	.21
Learning Slope: difference in exploratory errors (Trial 1-Trial 2)*	5.01 (.65)	1.10 (.78)	F(1,145) = 14.04, p<.001	.64
Learning Slope: difference in exploratory errors (Trial 3- Trial 5)	2.26 (.48)	2.69 (.57)	F(1,145) = .32, p = .57	.10
*Delayed Recall Trial*				
Exploratory Errors	7.53 (.46)	8.49 (.56)	F(1,145) = 1.66, p = .20	.22
Perseverative Errors	.74 (.13)	.63 (.15)	F(1,145) = .30, p = .58	.09
Rule-break errors	.36 (.08)	.31 (.10)	F(1,145) = .13, p = .71	.07
Savings score	−.55 (.61)	−1.71 (.73)	F(1,145) = 1.17, p = .28	.20

Note: Values adjusted for age, sex, and timed chase test scores.

*Groups differ, p<.05, aMCI = amnestic mild cognitive impairment.

**Figure 2:**
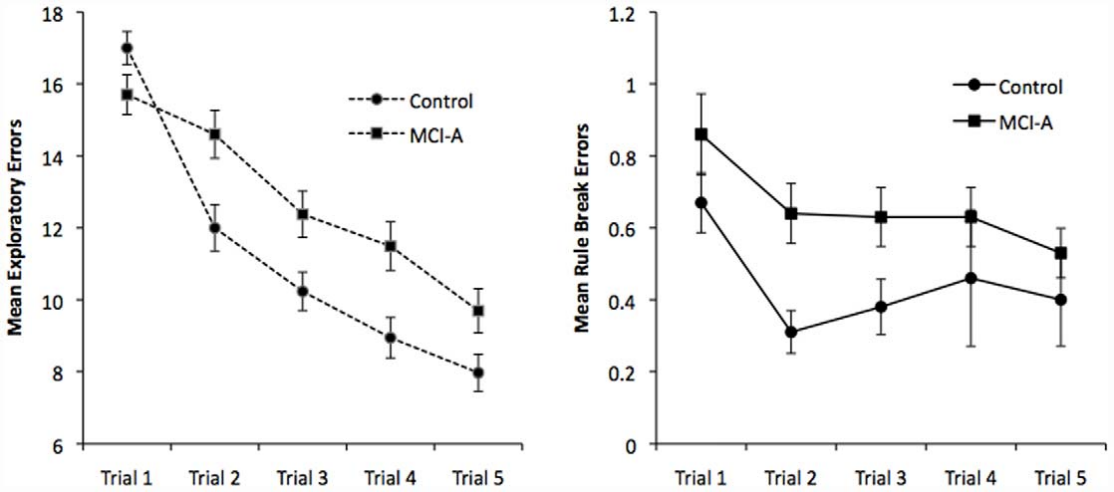
Mean Exploratory Errors and Rule Break Errors (with Standard Errors) over five learning trials of the Groton Maze Learning Test in Healthy Controls versus aMCI groups.

Figure 2 shows learning curves for total number of exploratory and rule-break errors on the GMLT for the aMCI and healthy control groups. The healthy control group showed a more substantial improvement in performance on the GMLT 2^nd^ learning trial, suggesting greater benefit from the first exposure to solving the hidden maze compared to the aMCI group. In the healthy control group, the slope of the learning curve (accuracy) over trials 1 to 2 was four times as steep as that observed for the aMCI group; however, the difference in slopes from trials 3 to 5 was not significant. A similar pattern of performance was observed for rule-break errors.

A MANCOVA of GMLT recall measures during the delayed recall trial did not reveal a significant effect of group [F(4,140) = .68, p = .60) or any of the other main effects of interactions [all F's(4, 140)<1.97, all p's>.10]. As shown in the bottom panel of Table 3, none of the delayed recall measures, including exploratory errors, perseverative errors, rule-break errors, and savings scores, differed by group.

## Discussion

The purpose of this study was to use a hidden maze learning task to analyze component processes of executive function and spatial learning dysfunction in a well-characterized sample of individuals with aMCI. Results of this study showed that, relative to healthy controls, individuals with aMCI made significantly more learning (exploratory) and working memory (rule-break) errors during the initial, acquisition trials of the task. We had hypothesized that the aMCI group would perform more exploratory errors given their primary amnestic syndrome, but found that they also made a significantly greater number of rule-break errors compared to healthy controls. This finding suggests that reductions in aspects of executive function (e.g., error monitoring) may in fact be present in a group otherwise identified as single-domain amnestic MCI using more conventional neuropsychological measures. These results further suggest that performance on a task of complex visuospatial executive function is compromised in individuals with aMCI, and likely explained by reductions in initial strategy formulation during early stages of visual learning.

Compared to the healthy control group, the aMCI group differed in the processes by which they learned the hidden maze path. In addition to having greater difficulty with initial strategy acquisition (i.e., making a greater number of rule-break errors), the aMCI group evidenced a substantially less steep learning curve from the first to second learning trial of the maze. Nevertheless, there was no statistically significant difference in mean errors between groups from the third to fifth learning trial, which indicates that while the aMCI group had difficulty in their initial acquisition strategy and developing an internal spatial map, this did not affect their ability to eventually learn the hidden pathway over later trials; this interpretation is further supported by the fact that the groups did not differ on any of the delayed recall measures. Given that the aMCI group scored lower but not in the impaired range compared tothe healthy control group on the ROCF-Copy (see Table 2), reduced visual organization may account for reductions in initial strategy acquisition and encoding of a hidden maze pathway on the Groton Maze Learning Test. Results of the current study extend previous research [14], [15], [16] to suggest that aMCI patients may have difficulty with the organization and initial encoding of visual information. Individuals with aMCI also made more exploratory and rule-break errors than healthy controls, with the magnitudes of the effect sizes of these group differences (d's = .41 and .21, respectively) comparable to a previous study of executive function in aMCI. For example, Brandt and colleagues [9] found that individuals with aMCI performed more poorly on tests of working memory (e.g., Trail Making Test Part B-A) and planning/problem solving (e.g., Porteus Maze), and that the magnitudes of these differences were moderate (i.e., d's = .49 and .46, respectively). Importantly, results of the current study suggest that employment of a cognitive test that yields several performance measures may yield greater insight into component visuospatial learning and executive processes that may be reduced in aMCI. For example, the most pronounced performance decrement observed in the current study was for the learning slope of exploratory/learning errors made over the first two learning trials (d = .64). This finding suggests that difficulties with efficient encoding—remembering the hidden pathway while following the rules—during early visual learning may account for reduced visual learning in individuals with aMCI.

The finding that the aMCI group made an increased number of errors reflecting both working memory (i.e., rule-break errors) and spatial learning (i.e., exploratory errors) processes is consistent with an earlier study of healthy older adults in which we observed relatively greater increases in errors reflecting these processes following low dose administration of the muscarinic acetylcholine antagonist scopolamine [21]. Results of this study suggested that scopolamine administration was associated with a pronounced (d>1.5) reduction in both the acquisition and application of the general rules (i.e. rule-break errors), spatial learning (i.e. exploratory errors), and error monitoring (i.e., perseverative errors) on the Groton Maze Learning Test. In the current study, individuals with aMCI made a greater number of exploratory and rule-break errors compared to healthy controls, but did not differ with respect to perseverative errors. Importantly, magnitudes of the group differences observed in the current study were much smaller than those observed in the scopolamine study. One explanation for this finding is that targeted muscarinic acetylcholine receptor antagonism with scopolamine gives rise to much more pronounced and widespread reductions in performance across all GMLT performance measures (d's>1.50), while individuals with aMCI may show less pronounced, albeit clinically meaningful, decrements in GMLT performance relative to controls (d's = .17 to .41). While additional research is needed to elucidate neurobiological changes that may account for these observed performance decrements in aMCI, results of this previous study that used scopolamine as a model of early AD suggests that reduced performance on GMLT measures of exploratory and rule-break errors may arise from disruption to cholinergic neurotransmission. The finding in the current study that the aMCI group made a greater number of rule-break errors compared with the healthy control group suggests that reduced performance on the GMLT may reflect difficulty in the acquisition and/or application of test rules, as well as in using information held in working memory to facilitate spatial learning and memory performance on this task.

The aMCI group did not evidence robust improvement in performance on the second trial of the hidden maze task compared to the healthy control group. Previous studies using the Groton Maze Learning Test have noted that the first learning trial is distinguished from all subsequent trials because for this first trial the hidden path is entirely unknown, as the participant is beginning to develop an internal spatial map of the hidden path by following the task rules and making exploratory errors [29], [30]. In fact, Mathewson and associates [29] showed differences between the first and subsequent learning trials in both the amplitude and latencies of the feedback-related negativity (FRN) potential, generated over the region of the anterior cingulate gyrus, when participants receive a signal that they have made an error. Given that the feedback for rule use is constant across all trials of the Groton Maze Learning Test and that the hidden path remains unchanged from trial to trial within a given test administration, the major difference in performance between the first and second trials is that on the second trial, individuals have been primed by a first exposure to the path, which must be held in working memory in order to successfully complete subsequent trials.

Neuroimaging studies suggest a possible neural basis for the findings observed in this study. For example, studies have shown that the hippocampus is active during the encoding of new spatial information [31] and that in individuals with aMCI, hippocampal activation may be reduced, with compensatory activation of anterior cingulate and medial frontal cortex [32]. For example, a positron emission tomography study found that while healthy controls evidenced predominantly left frontal activity and posterior cingulate activity during the encoding trials of an episodic memory task, and right frontal plus left temporal activity during retrieval, individuals with MCI did not demonstrate this pattern of activation and instead showed compensatory increases in activation in the occipital cortex during encoding and left frontal lobe during retrieval [33]. More research using neuromaging measures during the Groton Maze Learning Test performance is needed to examine the neural correlates of visuospatial executive function and learning on this task.

Neuroanatomical changes are seen relatively early in amnestic MCI patients in both the hippocampus and entorhinal cortex, which are associated with learning and integrating information [34]. Emerging research has also suggested that thickness in numerous regions of frontal cortex and bilateral posterior cingulate is associated with increased episodic memory performance above and beyond the contribution of the mesial temporal regions associated with episodic memory in individuals with MCI. Further, individuals with aMCI who scored low on measures of executive function showed greater cortical thinning in these same brain regions relative to those who scored higher on measures of executive function despite equivalent hippocampal volumes and thickness of mesial temporal lobe regions [17]. Taken together, these studies suggest a complex interplay between brain regions implicated in both executive function, and learning and memory in individuals with aMCI.

Although performance on the Groton Maze Learning Test is associated with hippocampal functioning similar to three-dimensional navigation tasks, this test is two-dimensional and does not require egocentric orienting. However, given the GMLT's association with a virtual reality navigation task [35] and other non-egocentric tasks such as block tapping and complex figure drawing [29], [30], it is important to further explore how the formation of internal pathways relates to formation of internal maps; this is particularly relevant in studies of aMCI and AD, given declines in hippocampal integrity, as well as learning and memory function.

Methodological limitations of this study must be noted. First, both a strength and limitation of this study is the well-characterized sample of individuals with aMCI. This represents a strength inasmuch as the highly screened sample ensures that comorbid medical, neurologic, and/or psychiatric conditions do not confound the observed associations; it represents a limitation, as it is not clear whether results of this study are generalizable to the broader population of individuals with aMCI, who often present with comorbidities [9]. Second, this study focused solely on characterizing component processes of visuospatial executive function and learning in individuals with aMCI. Whether results of this study differ in other subtypes of MCI, such as non-amnestic MCI or multi-domain amnestic MCI remains to be examined. Based on previous research, which has demonstrated more pronounced reductions in planning/problem solving and working memory in non-amnestic and multi-domain MCI [9], individuals with these subtypes of MCI may experience more difficulties in with the acquisition of novel and complex spatial information. Despite these limitations, results of the current study provide an example of how component visuospatial executive and learning processes involved in the performance of certain neuropsychological tasks may help develop a more refined understanding of the cognitive pathology of different types of MCI and ultimately of pre-MCI elderly at risk for dementia.
